# Characterizing cellular mechanical phenotypes with mechano-node-pore sensing

**DOI:** 10.1038/micronano.2017.91

**Published:** 2018-03-12

**Authors:** Junghyun Kim, Sewoon Han, Andy Lei, Masaru Miyano, Jessica Bloom, Vasudha Srivastava, Martha R. Stampfer, Zev J. Gartner, Mark A. LaBarge, Lydia L. Sohn

**Affiliations:** 1 Department of Mechanical Engineering, University of California at Berkeley, Berkeley, 94720-1740 CA USA; 2 Department of Bioengineering, University of California at Berkeley, Berkeley, 94720-1762 CA USA; 3 Department of Population Sciences, Beckman Research Institute, City of Hope, Duarte, 91010 CA USA; 4 Department of Pharmaceutical Chemistry, University of California, San Francisco, San Francisco, 94143 CA USA; 5 Biological Systems and Engineering Division, Lawrence Berkeley National Laboratory, CA, 94720 USA; 6 Graduate Program in Bioengineering, University of California, Berkeley, and University of California, San Francisco, Berkeley, 94720 CA USA

**Keywords:** Engineering, Microfluidics

## Abstract

The mechanical properties of cells change with their differentiation, chronological age, and malignant progression. Consequently, these properties may be useful label-free biomarkers of various functional or clinically relevant cell states. Here, we demonstrate mechano-node-pore sensing (mechano-NPS), a multi-parametric single-cell-analysis method that utilizes a four-terminal measurement of the current across a microfluidic channel to quantify simultaneously cell diameter, resistance to compressive deformation, transverse deformation under constant strain, and recovery time after deformation. We define a new parameter, the whole-cell deformability index (wCDI), which provides a quantitative mechanical metric of the resistance to compressive deformation that can be used to discriminate among different cell types. The wCDI and the transverse deformation under constant strain show malignant MCF-7 and A549 cell lines are mechanically distinct from non-malignant, MCF-10A and BEAS-2B cell lines, and distinguishes between cells treated or untreated with cytoskeleton-perturbing small molecules. We categorize cell recovery time, Δ*T*_r_, as instantaneous (Δ*T*_r_~0 ms), transient (Δ*T*_r_⩽40 ms), or prolonged (Δ*T*_r_>40 ms), and show that the composition of recovery types, which is a consequence of changes in cytoskeletal organization, correlates with cellular transformation. Through the wCDI and cell-recovery time, mechano-NPS discriminates between sub-lineages of normal primary human mammary epithelial cells with accuracy comparable to flow cytometry, but without antibody labeling. Mechano-NPS identifies mechanical phenotypes that distinguishes lineage, chronological age, and stage of malignant progression in human epithelial cells.

**Supplementary information:**

The online version of this article (doi:10.1038/micronano.2017.91) contains supplementary material, which is available to authorized users.

## Sensors: Microfluidic-based cell measurements could aid clinical diagnosis

A simple and innovative technique for measuring the mechanical properties of cells could lead to a versatile clinical diagnostic tool. The ability to measure differences in the mechanical properties of cells can be used to detect changes in cells that are caused by disease, aging, or environmental interactions. Present technologies for performing such measurements, however, can analyse only a few cells each hour. This led Lydia Sohn at the University of California, Berkeley, in the United States, and colleagues to use a microfluidic platform that integrates node-pore sensors with a contraction channel to measure mechanical differences in populations of cells efficiently. The team's device, which measures the current across the microfluidic channel and quantifies four biophysical properties of a single cell simultaneously, has broad applications for understanding biomechanical properties of cells, clinical diagnostics, and therapeutics.

**Supplementary information:**

The online version of this article (doi:10.1038/micronano.2017.91) contains supplementary material, which is available to authorized users.

## Introduction

Cells derive their mechanical properties from the structure and dynamics of their intracellular components, including the cytoskeleton, cell membrane, nucleus, and other organelles—all of which, in turn, emerge from cell type-specific genetic, epigenetic, and biochemical processes. The ability to identify differences within a population of one cell type or different cells among heterogeneous populations, or to detect changes due to disease or environmental interactions all based on cellular mechanical properties has potentially important implications for cell and tissue biology and clinical metrics. As examples, metastatic potential^1,2^, cell-cycle^3,4^, differentiation state^5–10^, the outcome of tissue self-organization^11^, and infection with intracellular pathogens^12,13^ have all been shown to correlate with changes in cellular. Even the process of aging has been shown to affect the ability of cells within the vascular system and musculoskeletal system to recover from mechanical deformation^14^. Thus, methods to measure multiple cellular mechanical properties rapidly and accurately have tremendous potential as label-free research tools and diagnostics.

Atomic-force microscopy (AFM)^15–17^ and micropipette aspiration^18,19^ are the gold standard for performing mechanical measurements on cells. These methods provide controlled loading conditions (for example, stress relaxation and creep indentation) and quantify such cellular properties as elastic modulus and cortical tension. They are, however, burdened by slow throughput, capable of analyzing only just a few cells per hour^7,20^, although recent adaptations of both methods have demonstrated higher throughput via more efficient analysis^21,22^. Likewise, optical tweezers^23,24^ and microplate rheometery^25^—two other well-established methods to measure cellular mechanical properties—also suffer from low throughput. Given these drawbacks, a number of microfluidic platforms have consequently been developed, including hydrodynamic stretching cytometry^26–28^, suspended microchannel resonators (SMR)^29^, and real-time deformability cytometry (RT-DC)^30^, to name only a few. Each of these methods, through optical imaging or measuring changes in resonant frequencies, can analyze populations of cells in a relatively short time (for example, 2000–65 000 cells per for hydrodynamic stretching cytometry^26–28^, 30 cells per s for SMR^29^, and 100 cells per s for RT-DC^30^). To identify specific cell types, these methods most often focus on correlating cell size or mass with a specific mechanical property. For example, hydrodynamic stretching cytometry and RT-DC compare cellular deformability with cell size, and SMR determines the transit time of cells through a narrow channel with respect to cell mass. Populations of cells are complex with respect to the continua of cell states that are represented within, and as such, multiple biophysical parameters are necessary to deconvolve and identify complex cellular mixtures. Recently, Masaeli *et al.*^31^ and Lin *et al.*^31,32^ have reported using deformability cytometry to measure multiple parameters, such as cell size, morphology, and relaxation rate, while cells undergo deformation. In so doing, they were able to identify different cellular states associated with pluripotent and neural stem-cell differentiation, respectively. While this achievement emphasizes the need for measuring multiple biophysical parameters to identify specific cell types, Masaeli *et al.*^31^ and Lin *et al.*^31,32^ focus on defining cellular phenotypes only while cells undergo deformation. Since overall recovery of a cell once released from deformation plays significant roles in cellular migration processes such as cancer metastasis^33^ and in providing a protective mechanism of cells against mechanical damage^34–36^, it is imperative for mechano-phenotyping platforms to have a temporal window sufficient enough to analyze the recovery that a cell undergoes after deformation.

Here, we describe a novel microfluidic platform called ‘mechano-Node-Pore Sensing’ (mechano-NPS). Mechano-NPS involves integrating a node-pore sensor^37,38^ with a contraction channel and performing a four-terminal measurement of the current across the integrated microfluidic channel to quantify four biophysical properties of a single cell, simultaneously: diameter, resistance to compressive deformation, transverse deformation, and recovery from deformation. This electronic-based method of multi-dimensional mechanical phenotyping provides the means to use these biophysical parameters as label-free biomarkers for identification and differentiation among cell types and, uniquely, to determine the effects of chronological age and malignant progression on cell elasticity and recovery from deformation. Mechano-NPS distinguishes malignant from non-malignant immortal epithelial cells and measures deformability changes in the cytoskeleton. In addition, mechano-NPS can discriminate between sub-lineages and among chronological age groups of primary normal human mammary epithelial cells (HMECs) based solely on their mechanical properties. Mechano-NPS represents an efficient, simple, and direct means to quantify multiple mechanical properties of single cells in heterogeneous populations.

## Materials and methods

### Experimental design

**Figure 1:**
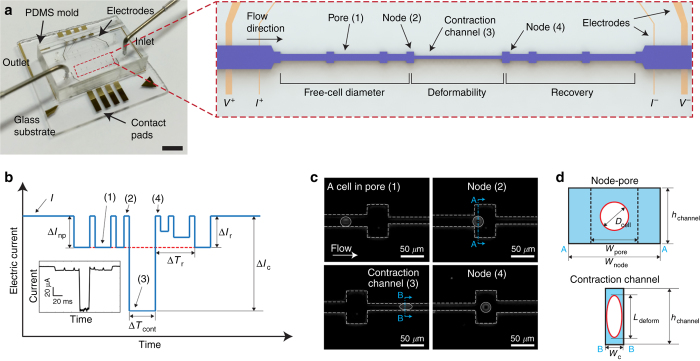
Principle of mechanical phenotyping via mechano-NPS. (**a**) A photographic image of the microfluidic platform. The scale bar corresponds to 4 mm. Red-dashed box shows a close-up view of the entire microfluidic channel. The microfluidic channel (pore) is segmented by nodes and a contraction channel. Two electrodes at both ends of the channel apply a constant voltage (1 V), and two inner electrodes measure the change of current across the channel. The regions where free-cell diameter, deformed diameter, and cell recovery are measured are as indicated. (**b**) Expected current pulse generated by a cell transiting the microfluidic channel. *I*, Δ*I*_np_, Δ*I*_c_, and Δ*I*_r_ correspond to the baseline current and the current drop by a cell transiting a node-pore, a contraction channel, and a node-pore after the contraction channel, respectively. Numbers in parentheses (1–4) correspond to the same specific segments of the microchannel (pore, node, and contraction channel) in (a). Δ*T*_cont_ corresponds to the time duration of a cell passing through the contraction channel, and ΔT_r_ indicates the time needed for Δ*I*_r_ to equal Δ*I*_np_ (see Supplementary Figure S6 for detailed information). (inset) An actual current pulse caused by a human mammary epithelial cell traversing the channel. (**c**) Time-snapshots of an MCF-7 cell (bordered by a white circle) in each of the different segments of the microfluidic channel (white dashed line; see Supplementary Videos 1 and 2 for detailed information). Numbers in parentheses (1–4) correspond to the same specific segments of the microchannel (pore, node, and contraction channel) in (a). (**d**) Cross-sectional diagram of the channel segments occupied by a cell. ‘AA’ and ‘BB’ indicate the corresponding cross-sections in (**c**). *w*_pore_, *w*_node_, w_c_, and *h*_channel_ correspond to the widths of the pore, node, and the contraction channel, and the height of the channel, respectively. *D*_cell_ and *L*_deform_ correspond to the free-cell diameter in the node-pore channel and the elongated length of the deformed cell in the contraction channel, respectively.

The platform consists of a 30 μm-high microfluidic channel embedded in a polydimethylsiloxane (PDMS) mold bonded to a glass substrate with pre-defined platinum (Pt) electrodes and gold (Au) contact pads (Figure 1a). The central part of the channel, which we refer to as the ‘contraction channel’, is long (2055 μm) and narrow (10 or 12 μm-wide) and flanked on either side by a series of nodes and pores that are 85 and 25 μm wide, respectively (Figure 1a, inset). The length of the contraction channel was chosen to provide sufficient time (~30 ms) over which a cell experiences constant applied strain. The node and pore dimensions were chosen for sufficient signal-to noise ratios. Given the flexibility and ease of device design and fabrication, different contraction channel lengths and node and pore dimensions could be employed. Filters that are 25 μm in width (the width chosen based on the size range of cells measured in these studies, aproximately 15–20 μm in diameter) are included at the entrance of the microfluidic channel in order to remove cellular clusters that may otherwise clog the device. Applying a constant DC voltage (1 V) across the channel, we employ a four-terminal measurement technique^37–40^ to measure the current pulses caused by cells transiting across the microfluidic channel when a non-pulsatile pressure of ~21 kPa (determined by a commercial pressure gauge, SSI Technologies) is utilized (Figure 1b and Supplementary Figure S1). After low-pass filtering all current versus time data, we employ custom-written software to extract both the magnitude and duration of each current sub-pulse (Δ*I*_np_, Δ*I*_c_, Δ*T*_cont_, and Δ*T*_r_ in Figure 1b) (Supplementary Figure S2).

Power analysis was employed to ensure that our sample size for mechanical phenotyping offers adequate power (≥0.80) to detect differences between experimental groups within a 95% confidence interval^41^ from the measured data set. For all cases which have a *P*-value<0.05, the analyzed sample size (*N*_a_) provided sufficient power value to measure statistical differences (Supplementary Table S1). Statistical significance was determined by performing a paired *t*-test or *χ*^2^-test. To ensure repeatability of results, all data presented in this study were measured using multiple microfluidic devices. The wCDI of MCF-7 cells obtained with different device replicas showed no statistical difference (Supplementary Figure S3, *P*=0.173).

### Device fabrication

To make the PDMS molds of our microfluidic platform, we employ standard soft-lithography. Briefly, we fabricate negative-relief masters onto polished silicon wafers. After mixing and degassing, we pour a 9:1 pre-polymer: curing agent mixture of PDMS (Sylgard 184, Dow Corning) onto the masters and subsequently cure them at 80 °C for 60 min. A slab of PDMS with the embedded microfluidic channel is excised from the master, and entry and exit ports are cored with a 1 mm diameter biopsy punch. To complete the device, we first expose the PDMS mold and a glass substrate with pre-defined electrodes to an oxygen plasma (470 mTorr, 80 W, 1 min), then align and mate the two together, and finally place the device onto a hotplate set to 80 °C for 60 min. For the specific surface-treatment experiments described, we injected either poly-D-lysine (PDL, 1 μg mL^−1^ in PBS) or bovine serum albumin (BSA, 2% w/v in PBS) into the completed device. After incubating for 2 h at 37 °C, we flushed the device with PBS and immediately began screening cells.

To fabricate the Pt electrodes and the Au contact pads onto glass substrates, we utilize standard photolithography for patterning. Using electron-gun evaporation, we deposit a 75/250/250 Å Titanium (Ti)/Pt/Au thin film onto the patterned substrates. We then use a gold wet etch (GOLD ETCHANT TFA, Transene Company) to expose the Pt electrodes.

### Cell culture

MCF-10A cells (ATCC CRL-10317) were cultured in MEBM medium, supplemented with 0.1% insulin, 0.1% hEGF, 0.4% hydrocortisone, and 10% cholera toxin. MCF-7 cells (ATCC HTB-22) were cultured in DMEM (Fisher Scientific, BW12719F), supplemented with 10% fetal bovine serum (FBS), 0.1 mM MEM Non-Essential Amino Acids (NEAA), 2 mM L-glutamine, and 1% Pen-Strep. BEAS-2B cells (ATCC CRL-9609) were cultured in BEGM BulletKit (Lonza, CC-3170). A549 cells (ATCC CRM-CCL-185) were cultured in F-12K medium (Fisher Scientific, MT10025CV), supplemented with 10% FBS and 1% of Pen-Strep. Jurkat cells (ATCC TIB-152) were cultured in RPMI 1640 medium (Thermo Fisher Scientific, Grand Island, NY USA), supplemented with 10% fetal bovine serum (FBS), and 1% Pen-Strep. All cell cultures were maintained at 37 °C in 5% CO_2_ and routinely passaged, per published protocols^42,43^, once they reached 80% confluence.

Cells were dissociated by treatment with 0.25% trypsin/EDTA for either 3 min (MCF-7 and A549 cells) or 5 min (MCF-10A and BEAS-2B cells) at 37 °C (Refs. 44–46), washed with the respective growth media, centrifuged at 0.2 RCF, and re-suspended at a concentration of ~20 000 cells per mL in PBS. To ensure cell viability, cells were injected into the prepared devices for screening immediately following re-suspension.

### Primary human mammary epithelial cells

Primary HMEC strains were generated and maintained as described previously^47,48^. HMECs were grown in M87A medium containing cholera toxin and oxytocin at 0.5 ng mL^−1^ and 0.1 nM, respectively. Details on the derivation and culture of these HMEC can be found at Human Mammary Epithelial Cell (HMEC) Bank Website^49^. Research was conducted under Lawrence Berkeley National Laboratory Human Subjects Committee IRB protocols 305H002 and 108H004, which allows for the use of HMEC samples for future scientific research.

### Pharmacological inhibition of cytoskeletal components

We disrupted actin polymerization with Latrunculin A and B (Enzo Life Sciences, Farmingdale, NY USA)^50^. Prior to deformability measurements, MCF-7 and MCF-10A cells were incubated with 2.5 or 5 μg mL^−1^ LatA or LatB in each cell’s respective growth medium for one hour at 37 °C and 5% CO_2_ (Refs. 29,51,52). Cells were then released from culture flasks with 0.25% trypsin/EDTA, rinsed once with PBS, centrifuged at 0.2 RCF, and re-suspended in PBS at a concentration of ~100 000 cells per mL. To confirm that actin polymerization was successfully inhibited after incubation, cells were fixed by 4% (w/v) paraformaldehyde in PBS for 15 min. They were then permeabilized with 0.1% Triton-X 100 (Sigma-Aldrich, St. Louis, MO, USA) in PBS for 5 min. Cell nuclei and F-actin were then counter-stained with 4’,6-diamidino-2-phenylindole (DAPI, Sigma-Aldrich, 10236276001) and rhodamine phalloidin (Thermo Fisher Scientific, R415), respectively, per manufacturer’s protocol, and then imaged with a Zeiss LSM710 confocal microscope.

### Discriminating cell types based on the whole cell deformability index (wCDI)

We derived a dimensionless parameter, which we refer to as the whole cell deformability index (wCDI), to distinguish cell populations based on mechanical phenotype. We assume a functional relationship among the biophysical parameters of a cell and fluid flow as follows,

(1)F(E,Dcell,hchannel,Uflow,ΔTcont,µ,Lc)=0

where *E, D*_cell_*, h*_channel_*, U*_flow_, *U*_c_, *μ, and L*_c_ correspond to elastic modulus, free cell diameter, height of the microfluidic channel, flow velocity within the node segment leading to the contraction channel, the transit velocity of cells in the contraction channel, fluid viscosity, and the length of the contraction channel, respectively. Three fundamental dimensions (*n=3*)—mass (*M*), length (*L*), and time (*T*)—are included in each of these six parameters (*n’=7*) as follows,

(2a)E=[ML−1T−2]

(2b)Dcell=[L]

(2c)hchannel=[L]

(2d)Uc=[LT−1]

(2e)Uflow=[LT−1]

(2f)µ=[ML−1T−1]

(2g)Lc=[L]

Following the Buckingham *π* theorem^53^, the relationship among these parameters can be written in terms of a set of four dimensionless parameters (*n′−n=4*). To find these dimensionless parameters (*π*_*i*_; *i*=1, 2, 3, and 4), we select repeating variables (*h*_channel_*, U*_flow_, and *μ*), where the number of required variables is equal to the number of fundamental dimensions (*n*=3). Multiplying one of the nonrepeating variables with the product of the repeating variables, we can define the following *π* terms,

(3a)π1=hchannelEUflowµ

(3b)π2=hchannelΔTcontUflow

(3c)π3=Dcellhchannel

(3d)π4=Lchchannel

We define the dimensionless parameter, wCDI (Equation (6)), to be the product of *π*_2_×*π*_3_×*π*_4_. The wCDI could also be defined as a function of *π*_1_, in which (*π*_1_=*f*(*π*_2_, *π*_3_, *π*_4_)), but the exact analytical expression can only be determined by experiment^53^. Comparing the wCDI with cellular cortical tension and the previously reported elastic modulus (*E*) of various cell lines (Supplementary Figure S4), we experimentally determined that the wCDI is inversely related to these traditional parameters.

### Cortical tension measurement using micropipette aspiration

Cortical tension was measured by micropipette aspiration as described previously^54,55^. Briefly, cells were trypsinized and resuspended in growth medium, and were transferred to the imaging chamber. Suction pressures in the range of 0.03–0.3 kPa were applied to the cells through an 8–10 μm glass micropipette. At each pressure, the cellular deformation inside the pipette was allowed to stabilize for 20–30 s before imaging. The average measurement from three images was used to calculate the length of deformation (*L*_p_). Subsequently, applied pressure was increased in 0.03 kPa increments till the *L*_p_ exceeded the radius of the pipette (*R*_p_). Any cell that blebbed was discarded. The critical pressure (*P*_crit_) is defined as the pressure at which the deformation inside the pipette is hemispherical, that is, *L*_p_*=R*_p_. The cortical tension (*T*_eff_) was then calculated using the following equation, where *R*_c_ is:

(4)ΔPcrit=2Teff×(1Rp−1Rc)

The cortical tension measurements from Jurkat, NIH 3T3, and HeLa cells are plotted from Schiffhauer *et al.*^56^.

## Results

### Population characterization of mechanical phenotypes at single-cell resolution

The repeated expansion and contraction of the width of our overall microfluidic channel shown in Figure 1a produces a unique and symmetric current pulse, consisting of sub-pulses, for each cell that transits the channel. Upon entering the microfluidic channel, a cell partially blocks the flow of current, and consequently, the measured current immediately drops from a baseline value, *I* (Figure 1b). When the cell enters the first node, the current returns to baseline only to drop again once the cell exits that node. This is a hallmark of node-pore sensing (NPS)^37,38^. The rise and fall of current repeats as the cell enters and exists the next two nodes. Upon entering the contraction channel where the width is narrower than the diameter of the cell, the cell deforms as shown in Figures 1c and d. Because the cell blocks nearly all of the current flow in this part of the channel, the current drop from baseline is far more dramatic than that resulting from the cell transiting the earlier pores (Supplementary Figure S5 and Supplementary Video 1). The cell subsequently enters and exits a series of node-pore pairs following the contraction channel, ultimately leading to the symmetrical shape of the overall current pulse. This symmetry is intentional by design and critically allows the monitoring of a cell’s recovery from constant strain deformation (Supplementary Video 2).

The magnitude of the current sub-pulse produced in the node-pore sequence (Δ*I*_np_) and the contraction channel (Δ*I*_c_) corresponds to the free-cell diameter (*D*_cell_) and cell elongation length (*L*_deform_), respectively (Figure 1d). The relationship among the current drop (∆*I)*, baseline current (*I*), particle diameter (*d*), the overall channel length (*L*), and the channel’s effective diameter (*D*_e_) is defined as^39,57,58^,

(5)ΔII=d3De2L[11−0.8(d/De)3]

To determine *D*_e_, we measure polystyrene microspheres of known size with the microfluidic channel (Supplementary Table S2). Using the values of ∆*I*/*I* arising from the microspheres, along with the known values of *L* and *d* (the size of the microspheres in this instance), we can numerically solve for *D*_e_ in Equation (5). Once *D*_e_ is known, we can subsequently determine *D*_cell_ of a screened cell by numerically solving for *d* in Equation (5) using the obtained values of Δ*I*_np_/*I*. We can also determine the volume of the deformed cell, *V*_deform_, by the relationship^39,57,58^, Δ*I*_c_/*I*~*V*_deform_/*V*_contraction_, where *V*_contraction_ is the volume of the contraction channel. To calculate *L*_deform_, we assume the cell undergoes an isometric deformation in the direction of both the channel’s longitudinal axis and channel height, resulting in an oblate-spheroid shape. From the relationship between the volume and major radius of the oblate spheroid, *V*_deform_=*πw*_c_*L*_deform_^2^/6 where *w*_c_ is the contraction-channel width, we can determine *L*_deform_ from Δ*I*_c_/*I*. We quantify the transverse deformation of the cell, *δ*_deform_=*L*_deform_*/D*_cell_, as it transits the contraction channel.

As a cell traverses through each section of the channel, the duration of the resulting sub-pulse produced by a cell corresponds to the cell’s transit time (Δ*T*) through that part of the channel. To quantify the resistance to compressive deformation, we utilize Δ*T*_cont_. To determine the recovery time of a cell from compressive deformation (Δ*T*_r_), we note the time required for the sub-pulses produced by the cell after exiting the contraction channel to return to the same shape and magnitude as those produced by the cell prior to entering the contraction channel, that is, when the cell returns to its original size and shape (Figure 1b and Supplementary Figure S6). Given the number of node-pore pairs and the overall length of the node-pore sequence we employ after the contraction channel, our device’s temporal window for measuring cell recovery is 40 ms. The flexibility of our device design and ease of fabrication allow for the inclusion of many more node-pore pairs after the contraction channel, which in turn would lead to an increase in time over which to observe recovery (Methods: Experimental Design). Based on all the recovery times we recorded with our particular device, we discriminate among three different cell-recovery types—instant (Δ*T*_r_~0 ms), transient (0<Δ*T*_r_⩽40 ms), and prolonged (Δ*T*_r_>40 ms) (Supplementary Figure S6).

Thus, from just a single current pulse produced by a cell transiting through the entire microfluidic channel, four biophysical properties of that cell—size (*D*_cell_), resistance to compressive deformation (Δ*T*_cont_), transverse deformation (*δ*_deform_), and recovery from deformation (Δ*T*_r_)—are extracted. These parameters are what we collectively use to mechanically phenotype a single cell, distinguish among cell types in a heterogeneous population, and determine subtle cellular changes.

### Distinguishing malignant and non-malignant epithelial cell lines based on mechanical phenotyping

**Figure 2:**
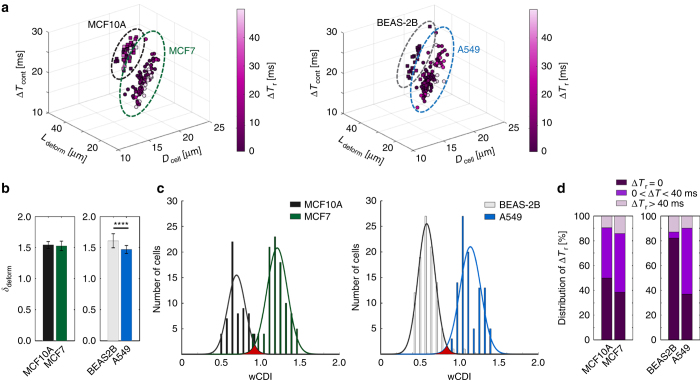
Mechanical phenotyping of malignant and non-malignant epithelial cells. (**a**) 4D plot of the free cell diameter (*D*_cell_), elongation length (*L*_deform_) due to an applied strain *ε*~0.3, transit time through the contraction channel (Δ*T*_cont_), and recovery time from compressive deformation (Δ*T*_r_) of malignant (MCF-7, *n*=99) and non-malignant (MCF-10A, *n*=99) breast cells, and malignant (A549, *n*=100) and non-malignant (BEAS-2B, *n*=100) lung cells. Dotted ovals group each cell line (MCF-10A: black, MCF-7: green, BEAS-2B: gray A549: blue). (**b**) Transverse deformation (*δ*_deform_) of MCF-10A, MCF-7, BEAS-2B, and A549 cells. Statistical differences were determined by a paired *t*-test (****P⩽0.0001). (**c**) wCDI distribution of MCF-10A, MCF-7, BEAS-2B, and A549 cells. Statistical differences were determined by a paired *t*-test. (MCF-10A vs MCF-7: *P*=3.90e-58, BEAS-2B vs A549: *P*=1.10e−80). The solid lines correspond to the fitted normal wCDI distribution for malignant and non-malignant cells, respectively. MCF-10A: $\overset{¯}{\mathrm{wCDI}}=0.699\pm 0.106$; MCF-7: $\overset{¯}{\mathrm{wCDI}}=1.230\pm 0.13$; BEAS-2B: $\overset{¯}{\mathrm{wCDI}}=0.590\pm 0.106$; and A549: $\overset{¯}{\mathrm{wCDI}}=1.151\pm 0.12$. (**d**) The proportion of cells screened that recovered instantaneously (Δ*T*_r_~0), required 40 ms or less (0<Δ*T*_r_⩽40 ms), or did not recover within the window of time measured (Δ*T*_r_>40 ms). A Chi-square test was employed to determine the statistical differences between the proportions of cell recovery types. There was no statistical difference in recovery types between MCF10A and MCF7 cells. In contrast, there was a significant statistical difference between BEAS-2B and A549 cells regarding instantaneous recovery (*P*⩽0.0001) and transient recovery (*P*⩽0.0001).

We investigated whether mechano-NPS could distinguish between immortal malignant and non-malignant states in two different epithelial tissue types based on their mechanical properties alone. We compared the mechanical properties of malignant MCF-7 with non-malignant MCF-10A breast epithelial cells and malignant A549 with non-malignant BEAS-2B lung epithelial cells when individual cells were subjected to a constant applied strain along the length of the contraction channel they traversed. Because strain, *ε*, is a function of both cell size and contraction channel width (*w*_*c*_), *ε*=(*D*_cell_−*w*_c_)/*D*_cell_, and prior independent measurement of *D*_cell_ showed that malignant MCF-7 and A549 cells are, on average, larger than non-malignant MCF-10A and BEAS-2B cells (Supplementary Table S3), we utilized a 12 μm-wide contraction channel to measure MCF-7 and A549 cells and a 10 μm-wide contraction channel to measure MCF-10A and BEAS-2B in order to achieve the same average *ε* (~0.3) for all cell types (Supplementary Table S3). As shown in the four dimensional (4D) graphs in Figure 2a, *D*_cell_ and *L*_deform_ of MCF-10A and BEAS-2B cells are significantly different from those of MCF-7 and A549 cells, respectively. Moreover, MCF-10A and BEAS-2B cells transit the contraction channel more slowly as compared to MCF-7 and A549 cells, respectively. When comparing transverse deformation (*δ*_deform_), we find that while A549 deform significantly less than BEAS-2B cells, MCF-7 and MCF-10A cells have similar deformation (Figure 2b).

Although our results clearly show that the transit time through the contraction channel (Δ*T*_cont_) is dependent on cell type (that is, malignant *vs*. non-malignant), so too could cell diameter affect transit time (Figure 2a)^59–61^. Because this could lead to difficulties in distinguishing cells within a heterogeneous population (Supplementary Figure S7), we employ the Buckingham *π*-technique^53^ to define a new dimensionless parameter, the whole-cell deformability index (wCDI), which relates *D*_cell_ and Δ*T*_cont_ by the following:

(6)wCDI=LcUflowhchannel⋅DcellΔTcont

where *U*_flow_ is the fluid velocity in the node section leading into the contraction channel, *L*_c_ is the length of contraction channel, and *h*_channel_ is the contraction-channel height (see detailed information in Methods: discriminating cell types based on the wCDI). *U*_flow_*, L*_c_ and *h*_channel_ are fixed values for any given experiment, and consequently, *D*_cell_ and Δ*T*_cont_ become the key parameters. Physically, the wCDI describes the deformability of the cell as a whole, including the cytoskeleton, nucleus, and organelles. Cells that are more deformable (that is, less stiff) transit through the contraction channel more easily, and subsequently at higher velocities, than those that are less deformable (that is, more stiff). Correspondingly, these cells will have a higher wCDI as compared to the latter, in accordance with Equation (6). Moreover, cells which are larger (smaller) will transit the contraction channel more slowly (quickly), and Equation (6) effectively negates this cell-size effect. While the Buckingham *π*-technique relates the wCDI to the cell’s elastic modulus, *E*, (see Methods, Equation (3a)), it does not define the explicit relationship between the two. We, therefore, performed side-by-side measurements of different cell lines (Jurkat, MCF-7, and MCF-10A) with the gold standard, micropipette aspiration, and also compared our measurements of MCF-7, MCF-10A, A549, and BEAS-2B cell lines with those obtained by AFM in the published literature^15,17,62–67^. Our results and subsequent analysis (Supplementary Figure S4) show that the wCDI is inversely proportional to both cortical tension and *E*, confirming our original physical description of the wCDI. While future studies are necessary to determine the exact analytical expression between the wCDI and *E*, mechano-NPS’s ability to mechanically phenotype cells successfully for cell-type discrimination is clearly demonstrated.

Figure 2c shows the wCDI distribution of non-malignant *vs*. malignant cells. The solid lines correspond to the fitted normal distribution of each population and the red-shaded region is the overlap area of the two distributions. As shown in Figure 2c, the wCDI of MCF-7 cells is significantly greater than that of MCF-10A cells with a 2.6% overlap. Similarly, A549 cells have a greater numerical wCDI than BEAS-2B cells, but with only a 1.6% overlap. Given the sensitivity demonstrated using the wCDI vs. Δ*T*_cont_ or cell size, alone (Figure 2c vs. Supplementary Figure S7), mechano-NPS and correspondingly the wCDI could potentially be utilized as a method for detecting subtle heterogeneities within cell populations such as those found in primary tissue^68,69^, heterogeneous cell lines and strains^70^, and biopsied tissue samples^71,72^.

Clear differences were observed in the recovery time after mechanical strain between breast and lung epithelial cell lines and, in the case of the latter, between malignant and non-malignant cell lines. Figure 2d shows that there was no statistical difference (using a Chi-square Analysis) regarding instantaneous recovery from mechanical deformation among breast epithelial cells (38.3% malignant MCF-7 cells vs. 50% MCF10-A cells, *P*=0.101). This is in striking contrast to lung epithelial cells in which there was a strong statistical difference (*P*<0.0001) between malignant and non-malignant cell lines: 37.0% of malignant A549 cells recovered instantaneously vs. 82.0% of non-malignant BEAS-2B cells screened (Figure 2d). Even though both are malignant cell lines, MCF-7 and A549 cell populations show surprising differences in their composition of transient and prolonged cell-recovery types. Whereas the majority of screened A549 cells transiently recovered (53.0%), MCF-7 cells were nearly evenly divided between transient and prolonged recovery (38.3% and 47.5%, respectively).

### Evaluating the contribution of cell-surface interactions and the cytoskeletal component, F-actin, to the mechanical phenotypes measured

**Figure 3:**
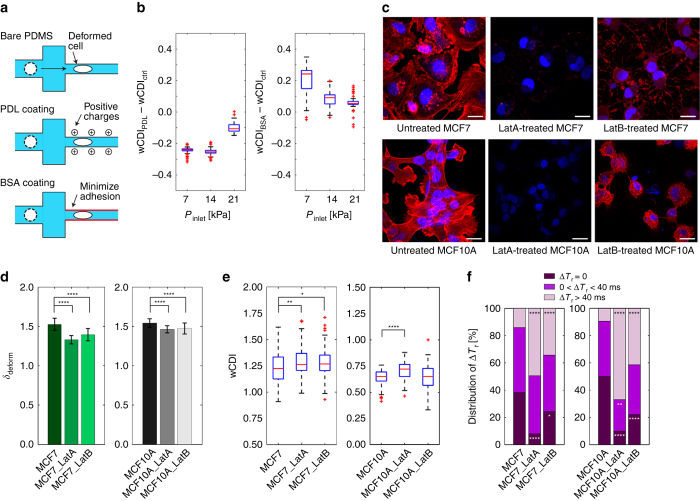
Contribution of cell-surface interaction and cytoskeletal component, F-actin, to the mechanical phenotypes of epithelial cells. (**a**) Schematic of experimental conditions used to measure the effects of cell-surface interaction on the wCDI. While poly-D-lysine (PDL) increases the positive charges on the channel wall for increased cell-surface interaction, bovine serum albumin (BSA) minimizes cellular adhesion to the channel wall. The control for all experiments was bare PDMS. (**b**) The difference in wCDI measured when MCF-7 cells transit a bare PDMS contraction channel (control) and a PDL-coated channel or a BSA-coated channel (*n*=99 for all cases) under various fluidic conditions. The difference in wCDI becomes smaller with greater *P*_inlet_. Within each box, the central red line corresponds to the median, and the edges of the box to 25% and 75% of the population. (**c**) Fluorescence images of MCF-7 and MCF-10A cells after treatment with Latrunculin A (LatA, 5 μg mL^−1^, 1 h) or Latrunculin B (LatB, 5 μg mL^−1^, 1 h). Cell nuclei and F-actin are stained with 4ʹ,6-diamidino-2-phenylindole (DAPI, blue) and rhodamine Phalloidin (red), respectively. Scale bar corresponds to 20 μm. (**d**) Transverse deformation (*δ*_deform_) of untreated, LatA-, and LatB-treated MCF7 and MCF10A cells (*n*=99). Statistical differences were determined by a paired *t*-test. (**e**) wCDI distribution of MCF-7 (*n*=99, Ctrl vs LatA: *P*=0.0074, Ctrl vs LatB: *P*=0.0253) and MCF-10A cells (*n*=99, Ctrl vs LatA: *P*=4.8940e-7, Ctrl vs LatB: *P*=0.9758), in which cells were either untreated or treated with LatA or LatB. Statistical differences were determined by a paired *t*-test. Within each box, the central red line corresponds to the median, and the edges of the box to 25% and 75% of the population. (**f**) The proportion of untreated and treated MCF-7 and MCF-10A cells screened that recovered instantaneously (ΔT_r_~0), required 40 ms or less (0<Δ*T*_r_⩽40 ms), or did not recover within window time measured (ΔT_r_>40 ms). The statistical differences between the proportions of recovery types of untreated and treated cells were evaluated by a Chi-square test. For all graphs, *, **, ***, and **** indicate *P*⩽0.05, *P*⩽0.01, *P*⩽0.001, and *P*⩽0.0001, respectively.

To determine whether cell-surface interactions greatly affect the passage of a cell within the contraction channel, and in turn contribute significantly to its wCDI, we screened MCF-7 cells in channels coated with either PDL or BSA and compared the resulting wCDI with that obtained by screening with a bare-PDMS channel (Figure 3a). PDL increases cell-surface interactions by adding positive charges on the PDMS channel walls^73,74^ and would therefore lead to a lower wCDI. In contrast, BSA inhibits cellular adhesion to the PDMS surface^75^ and would result in a higher wCDI. Figure 3b compares the wCDI obtained when MCF-7 cells were measured with bare-PDMS and PDL- and BSA-coated channels at different inlet pressures, that is, flow speeds. At low pressures (*P*_inlet_=7 kPa and 14 kPa), the average wCDI is appreciably lower in the PDL-coated channel and higher in the BSA-channel as compared to the bare-PDMS control channel. At *P*_inlet_=21 kPa, the inlet pressure at which we performed all our experiments, cells flow at a sufficiently high enough rate that cell-surface interactions are minimized within the contraction channel. As shown in Figure 3b, the obtained wCDI at this inlet pressure for either the PDL- or BSA-coated channel is not a dramatic shift from that measured with the bare-PDMS control channel. Moreover, the difference in wCDI among the different surface treatments vs. the bare-PDMS control channel at 21 kPa inlet pressure is significantly less than that measured between malignant and non-malignant epithelial cell types (Figure 2b). We, therefore, conclude that while surface-interactions do contribute to the wCDI, they are not the dominant factor at the higher inlet pressures or flow rates used for these studies.

Because we propose that mechano-NPS distinguishes cells based on mechanical differences, we should detect cytoskeletal perturbations. Thus, we treated MCF-7 and MCF-10A cells with the actin polymerization inhibitors, Latrunculin A (LatA) or B (LatB) (Figure 3c), and subsequently screened them under a strain magnitude, *ε*_avg_~0.3. We found that the cellular deformation in the transverse direction (*δ*_deform_) of both MCF-7 and MCF-10A cells treated with LatA and LatB was significantly reduced compared to their respective controls (Figure 3d), with MCF-7 cells generally more so than MCF-10A cells. Furthermore, we found that the wCDI increased for both LatA- and LatB-treated MCF-7 cells, and for LatA-treated MCF-10A cells as compared to the untreated control cells (Figure 3e). In subsequent experiments, we observed that the change in wCDI caused by LatA treatment correspondingly increased with concentration for both MCF-7 and MCF-10A cells, with the latter more sensitive to the treatment (Supplementary Figure S8). This is in contrast, however, to no detectable change in wCDI of MCF-10A cells no matter the LatB concentration. Overall, the different response of MCF-7 and MCF-10A cells to LatA and LatB may be due to differences in F-actin content, but further experiments are warranted here. As we confirmed with staining and confocal microscopy that the F-actin filaments were indeed inhibited in the Lat A- and B-treated cells (more so with Lat-A than with Lat-B as shown in Figure 3c), we conclude that mechano-NPS successfully detects cytoskeletal perturbations induced by exogenous chemicals.

While differences between the wCDI of LatA-treated cells are more pronounced with MCF-10A cells than MCF-7 cells, the differences in recovery time for Lat A- and LatB-treated cells in both cell types vs. the control are far more significant. Figure 3f shows that Latrunculin treatment results in the slow recovery of both MCF-7 and MCF-10A cells from the sudden relief of deformation. Moreover, there is a statistically significant difference between untreated and treated cells regarding recovery. In the case of MCF-7, only 8.1% of LatA-treated and 24.2% of LatB-treated cells instantaneously recover vs. 38.3% of untreated cells. For MCF-10A, the majority of LatA- and LatB-treated cells (66.7% and 41.4%, respectively) do not recover within the 40 ms time window our device offers (vs. 9.7% of untreated control cells). As we also found, the changes in cellular recovery are generally more pronounced at higher concentrations of Latrunculin treatment (Supplementary Figure S8). These results support the notion that actin filaments contribute to the ability of cells to retain their original shape^36,76^. Moreover, mechano-NPS detects differences in recovery from deformation, either transiently or not at all, between LatA- and LatB-treatment that are consistent with LatA being the more avid inhibitor of actin polymerization.

### Mechanical phenotyping of human mammary epithelial cells

**Figure 4:**
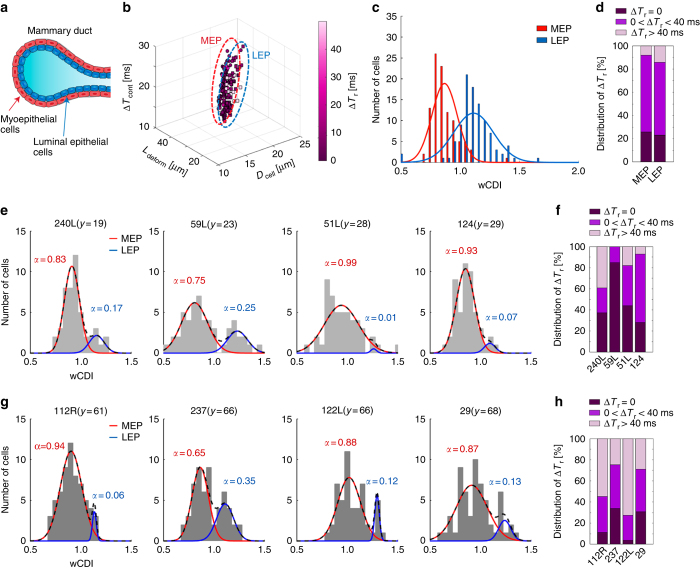
Mechanical phenotyping of HMECs. (**a**) Cellular structure of the human mammary gland. The mammary duct consists of an outer layer of myoepithelial cells (red) that surround an inner layer of luminal epithelial cells (blue). (**b**) 4D plot of the cell diameter (*D*_cell_), elongated length (*L*_deform_), transit time through the contraction channel (Δ*T*_cont_), and recovery time (Δ*T*_r_) of myoepithelial (MEP, *n*=99) and luminal epithelial (LEP, *n*=104) breast cells. Dotted ovals group each sub-lineage (MEP: red and LEP: blue). Pre-sorted MEP and LEP cells were screened with an applied strain magnitude *ε*~0.4. (**c**) wCDI distributions of MEP and LEP lineages (*P*=1.2047e-25). Statistical differences were determined by a paired *t*-test. The red and blue lines correspond to the fitted normal distribution of MEP ($\overset{¯}{\mathrm{wCDI}}=0.865\pm 0.107$) and LEP ($\overset{¯}{\mathrm{wCDI}}=1.133\pm 0.144$) cells, respectively. The wCDI overlap between the two lineages is 29.3%. (**d**) Distribution of pre-sorted MEP and LEP cells that have instant (*ΔT*_r_ ~0,), transient (0<*ΔT*_r_⩽40 ms), or prolonged (*ΔT*_r_>40 ms) recovery. (**e** and **g**) wCDI distribution of HMECs derived from young (**e**, y=age, 240L; *n*=54, 59L; *n*=53, 51L; *n*=50, 124; *n*=54) and old women (**g**, 112R; *n*=62, 237; *n*=59, 122L; *n*=54, 29; *n*=60). Outliers over 3 standard deviation of the mean were removed. The black dashed line corresponds to the fitted normal distribution of HMEC cells (MEP+LEP). The red and blue solid lines represent the normal distribution of MEP and LEP cells, respectively, with the ratio (*α*) of each lineage in the HMEC population as determined by the Expectation-Maximization algorithm^77^. (**f** and **h**) The proportion of HMECs from young (**e**) and old (**g**) women that have instant, transient, or prolonged recovery from applied strain.

To determine whether our platform could discriminate different lineages within a population of primary epithelial cells, we screened the mechanical phenotypes of HMECs, which broadly consist of two lineages: myoepithelial (MEP) cells and luminal epithelial (LEP) cells (Figure 4a). MEP and LEP cells have distinct roles in breast tissue. MEP cells play active roles in ductal contraction and in tumor suppression, and LEP cells produce milk and may represent a target-cell-type for carcinogenesis^78^. Previous studies of mammary epithelia have implicated profound roles of cytoskeletal components in morphogenesis^11,79,80^. We measured the mechanical characteristics of these two lineages of cells. Since both MEP and LEP cells have a similar size range (Supplementary Figure S7), we employed a 10 μm-wide contraction channel, corresponding to an *ε*_avg_~0.4 for all measurements. Figure 4b shows the relationship among the measured parameters of MEP and LEP cells (derived from a 66-year old woman, strain 237) that were FACS-enriched ahead of mechano-NPS characterization. Although LEP cells, on average, had a similar transverse deformation as that of MEP cells, they required less time to pass through the contraction channel (Figure 4b), thus suggesting that they are more deformable to an applied strain in the channel-width direction. Furthermore, while the deformed diameter and transit time of both lineages are dependent on the free cellular diameter, there are clear differences between the wCDI distribution of MEP ($\overset{¯}{\mathrm{wCDI}}=0.865\pm 0.107$) and LEP ($\overset{¯}{\mathrm{wCDI}}=1.133\pm 0.144$) cells (Figure 4c). In terms of cell recovery, MEP and LEP cells show a similar distribution of recovery types (Figure 4d).

We also measured the mechanical properties of primary HMEC cultures that consisted of mixtures of MEP and LEP cells from eight women of different chronological age (four pre-menopausal women aged <30 years and four post-menopausal women aged >55 years). Using the Expectation-Maximization algorithm^77^, in which the wCDI distribution function of sorted MEP and LEP cells obtained in our earlier experiments (Figure 4c) were used as initial values, we determined the ratio (*α*) of MEP and LEP cells within each primary HMEC strain (Figures 4e and g) and subsequently compared this ratio to FACS analysis of CD10+/CD227− MEP and CD10−/CD227+ LEP (Supplementary Figure S9). The component ratios of MEP and LEP cells, as determined by the wCDI distributions, match exceptionally well with those obtained from FACS, as confirmed by a Chi-square test with a *P*-value=0.05 (Supplementary Table S4). Indeed, the two methods are statistically indistinguishable. Although age-dependent differences in wCDI were not detected, age-dependent differences were readily apparent in recovery. Figures 4f and h show the composition of cell-recovery type for MEP and LEP cells of the young and old HMEC strains. Younger HMEC strains strikingly have a higher proportion of cells that recover instantaneously (an average of 47.8%) as compared to older strains (an average of 19.9%), suggesting that the cytoskeleton in younger cells is more resilient or more active, and in turn more responsive, to mechanical deformation.

**Figure 5:**
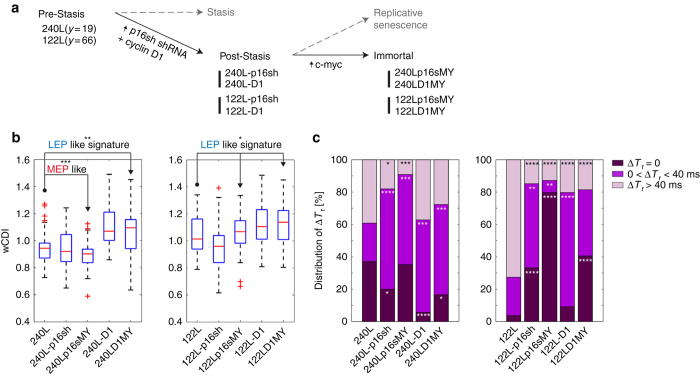
Mechanical phenotyping of HMECs undergoing immortalization. (**a**) The stages of malignant progression in breast epithelia. (**b**) wCDI distribution of HMECs per the outlined immortalization steps when cells were screened with an applied strain of *ε*~0.4 (*n*=54 for all cases) Compared to primary cells (240L and 122L, respectively), each population has following *P* values, 240L-p16sh: *P*=0.5306, 240Lp16sMY: *P*=0.0003, 240L-D1: *P*=0.0005, 240LD1MY: *P*=0.0094, 122L-p16sh: *P*=0.0205, 122Lp16sMY: *P*=0.5668, 122L-D1: *P*=0.023, and 122LD1MY: *P*=0.011. Statistical differences were determined by a paired *t*-test. Within each box, the central red line is the median, the red cross is an outlier, and the edges of the box correspond to 25 and 75% of the population. (**c**) Distribution of instant (Δ*T*_r_~0,), transient (0<Δ*T*_r_⩽40 ms), or prolonged (Δ*T*_r_>40 ms) recovery within each HMEC population per immortalization step. The statistical differences between the proportions of recovery types of primary cells and each stage of malignant progression were evaluated by a *χ*^2^ test. For all graphs, *, **, ***, and **** indicate *p*⩽0.05, *p*⩽0.01, *p*⩽0.001, and *p*⩽0.0001, respectively.

We next determined whether HMEC traversing the stages of malignant progression have distinctive mechanical signatures that could be used to track these stages. We previously reported a method for producing post-stasis and immortal HMEC cell lines in the absence of gross, and confounding, genomic errors^81^. In this experiment, expression of p16 shRNA or cyclin D1 was used to bypass the stress-induced stasis barrier, and expression of c-myc was used to bypass the replicative senescence barrier and generate immortal non-malignant cell lines (Figure 5a). We used mechano-NPS to generate wCDI profiles and the recovery-type distribution of primary normal HMEC strains (240L and 122L), post-stasis finite strains (240L-p16sh, 240L-D1, 122L-p16sh, 122L-D1,), and immortal non-malignant cell lines (240Lp16sMY, 240LD1MY, 122Lp16sMY, 122LD1MY). Each stage of malignant progression had a unique wCDI distribution. 240LD1MY, 122LD1MY, and 122Lp16sMY are known to have molecular and biochemical signatures of the luminal cancer subtype^82^. Their wCDI profiles show a mean that is greater than those of their normal isogenic HMEC predecessors, which also is consistent with a more LEP phenotype (Figure 5b). In contrast, 240p16sMY have a molecular and biochemical phenotype of basal breast cancers, which bear more similarity to MEP than to LEP lineage, and the wCDI distribution was more consistent with that of MEP (Figure 5b). The post-stasis finite strains exhibited wCDI distributions that were intermediate phenotypes between normal HMEC and the isogenic immortal malignant cell lines, in a manner consistent with the eventual intrinsic luminal- or basal-like subtype of the immortal lines (Figure 5b). Interestingly, all immortal non-malignant cell lines screened have a greater fraction of cells that exhibit instant or transient recovery as compared to those of post-stasis finite strains (Figure 5c). When comparing the older pre-stasis strain, 122L to the isogenic immortal cell lines, there was a particularly stark decrease in recovery time (Figure 5c). Thus, we observed two different types of mechanical signatures: wCDI differed between the MEP and LEP lineages, whereas recovery from deformation was a distinguishing characteristic of chronological age. Moreover, these data provide functional evidence to suggest that the process of immortalization is associated with fundamental changes in the ability of cytoskeletons to respond to deformation.

## Discussion

Mechano-NPS is a versatile technique that can analyze populations of single cells for a number of biophysical properties, simultaneously. Our newly defined dimensionless parameter, wCDI, which corresponds to whole-cell deformability, allows us to compare different cell types directly. Complementing the wCDI, the quantification of the cellular deformation in the transverse direction when cells are subject to compressive deformation, cell recovery from deformation, and the subsequent distribution of different cell-recovery types provide unique information about a cell population. Utilizing just these three parameters, we have shown stark differences between, and even patterns of cell recovery among, malignant and non-malignant cells, sub-lineages and chronological age groups, along with changes in the cytoskeleton. In general, the multi-variable phenotyping achieved by mechano-NPS provides a comprehensive understanding of single-cell mechanical behavior. Hierarchical clustering analysis of the mechano-NPS-screened mechanical phenotypes demonstrates a relationship among specific mechanical phenotypes with respect to different cell lines and with respect to the malignant progression of HMECs (Supplementary Figures S11 and S12). In future studies, single-cell level mechano-profiling should enable the identification of rare and/or masked sub-populations that comprise a bulk cell population, as well as characterization of cell states during dynamics processes—not just those studied here—solely based on mechanical phenotype.

While we have focused on the wCDI, transverse deformation, and cell recovery here, additional biophysical parameters could be measured with mechano-NPS simply by adding more node-pore sequences, which would, for instance, increase the time resolution needed for investigating the mechanical plasticity of cells. We could also utilize different contraction channel geometries. For example, employing a sinusoidal contraction channel would induce periodic deformation to probe cellular viscoelastic properties, which depend non-linearly on the frequency of deformation. Taken together, the many biophysical properties that could be measured with mechano-NPS would lead to a better understanding of the origins of specific cellular mechanical properties and the mechanical contributions of different cellular components (for example, cytoskeleton, nuclear envelope, organelles, and their own associated non-linear properties). In general, however, mechano-NPS in its present form successfully mechanically phenotypes cells for identification. Additional attractive features of mechano-NPS include that it is label-free, screened cells remain viable (Supplementary Figure S12), and the potential to couple this technique with microfluidic cell-sorting technologies. We screened up to 350 cells per min with our mechano-NPS device in the experiments we have presented. Because of the overall length of the channel, coincidence events, in which more than one cell occupies the channel at any given time, occur on occasion, especially when screening a high concentration of cells. Because of their complexity, current pulses arising from these events are presently removed from analysis. Implementing advanced signal processing, such as match filtering, could deconvolve these particular pulses and substantially increase throughput by enabling higher flow rate and higher concentration of cells^83,84^. Although it currently has significantly lower throughput compared to hydrodynamic stretching cytometry^27^, deformability cytometry^31,32^, and RT-DC^30^, mechano-NPS does not rely on optical imaging and therefore can easily be scaled up. Many mechano-NPS channels can be operated in parallel, resulting in overall increased throughput (potentially on the order of many thousands of cells per min), while importantly still maintaining the ability to examine cell recovery. Equally important, the simplicity of mechano-NPS, even in multiplexed form, is preserved.

Mechanical phenotyping of cells is a nascent and active area of research. Cellular mechanical properties can reflect malignancy of cancer cells and their metastatic potential^85^. Using mechano-NPS and various other different methods to measure cell-to-cell mechanical properties opens up new possibilities to understanding the biological underpinnings of the different measurements. Mechano-NPS reveals and quantifies emergent functional properties of the cytoskeleton of cells. Consequently, mechano-NPS can evaluate cytoskeleton-targeted drugs (for example, estramustine, colchicine, and paclitaxel), which are often employed in cancer therapies^86,87^, and may provide a new window into drug resistance of cancer cells, which could be caused in part by their cytoskeletal components^88,89^. The ability of our platform to rapidly characterize mechanical properties in populations of cells lends itself to numerous applications in cell biology and basic research. For example, mechano-NPS could be used to assay rapidly common laboratory cell lines cultured under different conditions and confluences, and to determine whether cells coming out of culture are in a similar state from day-to-day. Clinically, mechano-NPS may yield a new approach to early detection of breast and other types of cancer genesis through analyzing epithelial cells and their composition ratio. Indeed, we have already demonstrated mechano-NPS’s ability to distinguish between LEP and MEP lineages in mixed populations, between epithelial cells from pre- or post-menopausal women, and between normal and immortal transformed epithelial cells from the same individual. The proportions of MEP and LEP subpopulations in mammary epithelium is highly associated with age of women^47^, and when combined with distinct deformation recovery phenotypes in normal and transformed cells, mechano-NPS may yield valuable information regarding risk or diagnosis of breast cancer. We previously reported that the intrinsic subtype of immortal transformed HMEC was observable at the earliest stage of progression, bypass of stress-induced stasis, using molecular and biochemical markers of lineage. Here, we show that the stage of progression and the intrinsic subtypes are associated with distinctive mechanical phenotypes, opening up the possibility that wCDI could be used in a diagnostic setting as well.

## Conclusion

Mechano-NPS is a multi-parametric, electronic-based, single-cell analysis method that can quantify cell diameter, resistance to compressive deformation, transverse deformation under constant strain, and recovery time after deformation, simultaneously. As demonstrated, the newly defined index wCDI, transverse deformation, and recovery time provide a quantitative mechanical metric for discriminating among different cell types, identifying sub-lineages of primary human mammary epithelial cells, and analyzing phenotypes that correlate with chronological age and malignant progression of human mammary epithelial cells. Mechano-NPS thus has great potential to be utilized as an efficient, label-free mechanical phenotyping tool for basic and clinical applications requiring characterization of cellular mechanics at the single-cell level.

## Supplementary information

Supplementary Information (PDF 9151 kb)

Supplementary Video S1 (MPG 117 kb)

Supplementary Video S2 (MPG 289 kb)
