# Supplementary material for: Characterizing cellular mechanical phenotypes with mechano-node-pore sensing
Source: Microsyst Nanoeng. 2018 Mar 12;4:17091. doi: 10.1038/micronano.2017.91 (PMC5958920; doi:10.1038/micronano.2017.91)
Supplement: Supplementary file 1 — Supplementary Information (PDF 9151 kb) [file 41378_2018_BFmicronano201791_MOESM6_ESM.pdf]

OPEN

Supplementary File

Characterizing cellular mechanical phenotypes with mechano-node-pore sensing

Junghyun Kim[1], Sewoon Han[1], Andy Lei[2], Masaru Miyano[3], Jessica Bloom[3], Vasudha Srivastava[4], Martha R. Stampfer[5], Zev J Gartner[4,6], Mark A. LaBarge[3,5] and Lydia L Sohn[1,6]

Microsystems & Nanoengineering (2018) **4,** 17091; doi:10.1038/micronano.2017.91; Published online: 12 March 2018

Supplementary Video 1. Cellular deformation at the contraction channel. Deformation of a MCF-7 cell entering the 12 μm-wide contraction channel. The cell was driven by non-pulsatile pressure (~21 kPa) at the inlet of the channel. Video was recorded with 1000 fps.

Supplementary Video 2. Cellular recovery once released from the deformation. Recovery of a MCF-7 cell releasing from the 12 μm-wide contraction channel. The cell was driven by non-pulsatile pressure (~21 kPa) at the inlet of the channel. Video was recorded with 1000 fps.

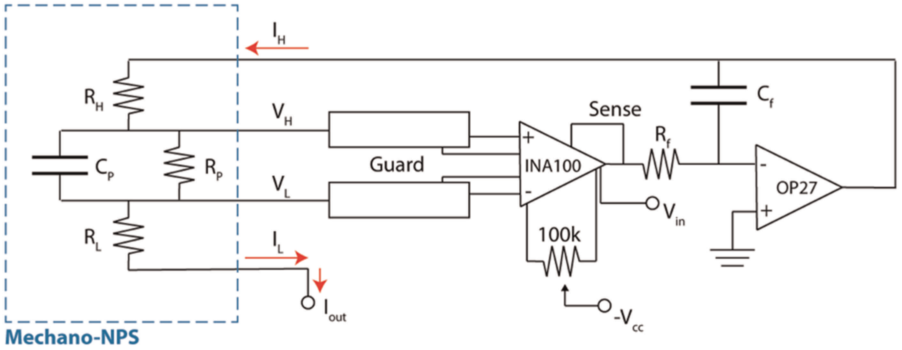

Figure S1 The electrical circuit model for the constant voltage four-point measurement[1,2]. The blue-dashed box represents the impedances of the electrode, fluid, and cell of the microfluidic device (Mechano-NPS). R_f and C_f represent the circuit elements. R_p and C_p indicate the resistance and capacitance of the microfluidic channel and R_H and R_L represent the resistance of the fluid in the inlet and outlet reservoir. Through the first amplifier (INA100), the output is a sum of the voltage difference across the microfluidic channel ($V_H - V_L$) and the input voltage, V_{in}. This becomes the inverting input for the second amplifier (OP27). Through this feedback arrangement, the current flows from I_H to I_L and then to I_{out} (red arrows).

[1]Department of Mechanical Engineering, University of California at Berkeley, Berkeley, CA 94720-1740, USA; [2]Department of Bioengineering, University of California at Berkeley, Berkeley, CA 94720-1762, USA; [3]Department of Population Sciences, Beckman Research Institute, City of Hope, Duarte, CA 91010, USA; [4]Department of Pharmaceutical Chemistry, University of California, San Francisco, San Francisco, CA 94143, USA; [5]Biological Systems and Engineering Division, Lawrence Berkeley National Laboratory, CA 94720, USA and [6]Graduate Program in Bioengineering, University of California, Berkeley, and University of California, San Francisco, Berkeley, CA 94720, USA.
Correspondence: Lydia L. Sohn (sohn@berkeley.edu)

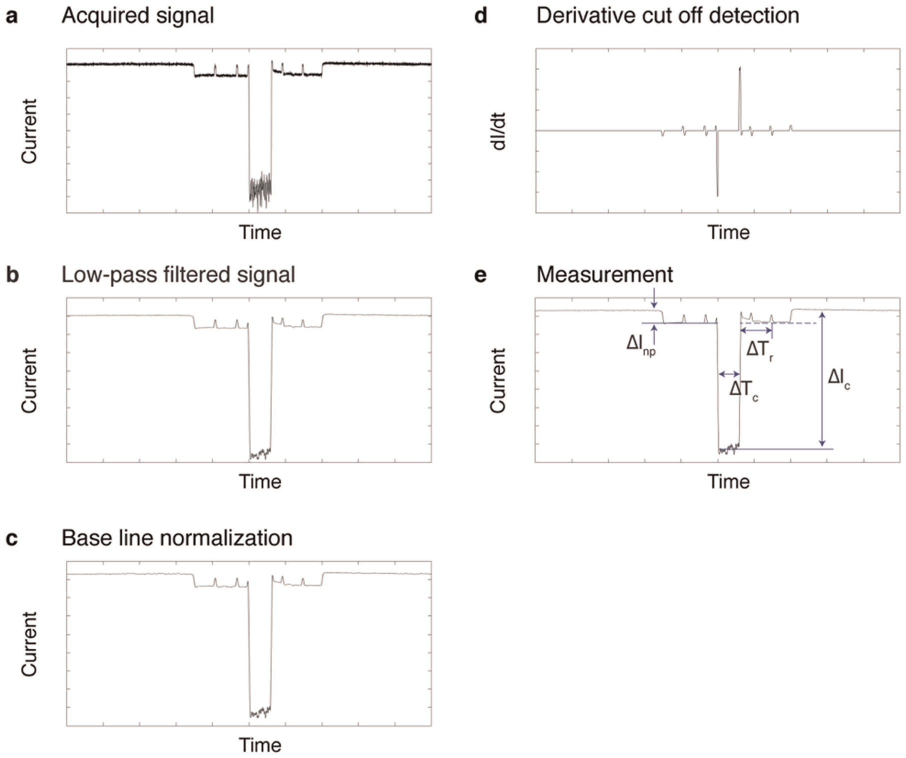

Figure S2 Signal processing by customized MATLAB code. The acquired signal (**a**) is first low-pass filtered (**b**) to remove noise. The base-line is then normalized (**c**) to remove any drift. (**d**) A derivative cut-off detection is subsequently employed as an index to determine the start and end point of each pulse. (**e**) Finally, the current pulse magnitude and duration are measured based on this index.

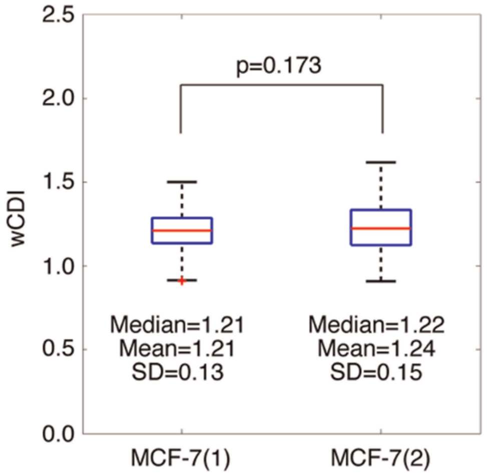

Figure S3 wCDI of MCF7 cells from different replicas of the mechano-NPS device. MCF7 cells were measured by different replica of the device showing no statistical difference (MCF-7(1): $n = 97$, MCF-7(2): $n = 99$, $P = 0.173$). The statistical difference was determined by a paired *t*-test. Within each blue box, the central line is the median and the edges of the box correspond to 25% and 75% of the wCDI distribution.

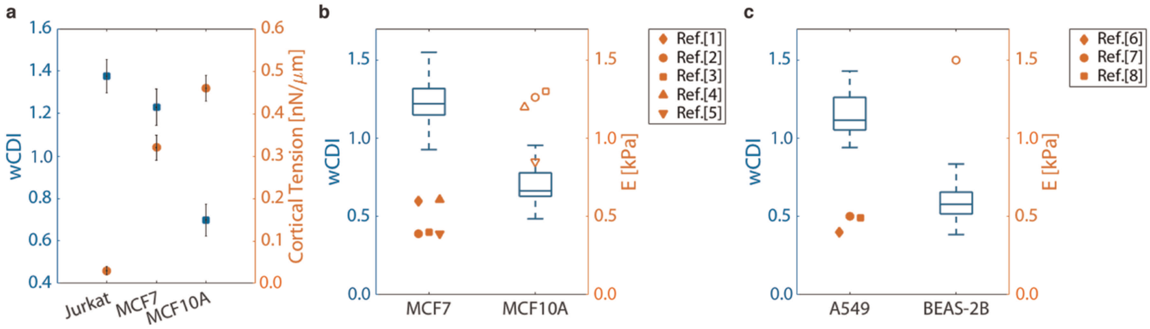

Figure S4 Relationship between mechanical properties and wCDI. (**a**) Comparison of wCDI with cortical tension as determined by micropipette aspiration of Jurkat, MCF7, and MCF10A cells. The wCDI is inversely related to cortical tension. Error bar indicates standard deviation for wCDI and standard error for cortical tension. (**b** and **c**) Comparison of wCDI with the elastic modulus, as measured by AFM, of breast cell lines (**b**) and lung cell lines (**c**). Within each blue box, the central line is the median and the edges of the box correspond to 25% and 75% of the wCDI distribution. The orange symbols are the reported elastic modulus of each cell line[3–10]. The trend of wCDI over various cell lines is inversely proportional to the elastic modulus.

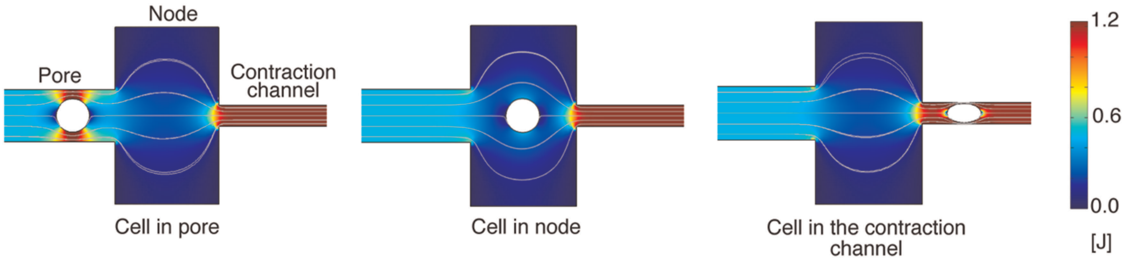

Figure S5 Computational modeling of the electric field when a cell transits each section of the mechano-NPS microfluidic channel. The fine lines correspond to the calculated electric-field lines in each section of the microfluidic channel, and the white circle corresponds to a cell. As determined, the electric-field density, ***J***, in the contraction channel is greater than that in the node. Computational simulation was performed using Comsol Multiphysics 5.0.

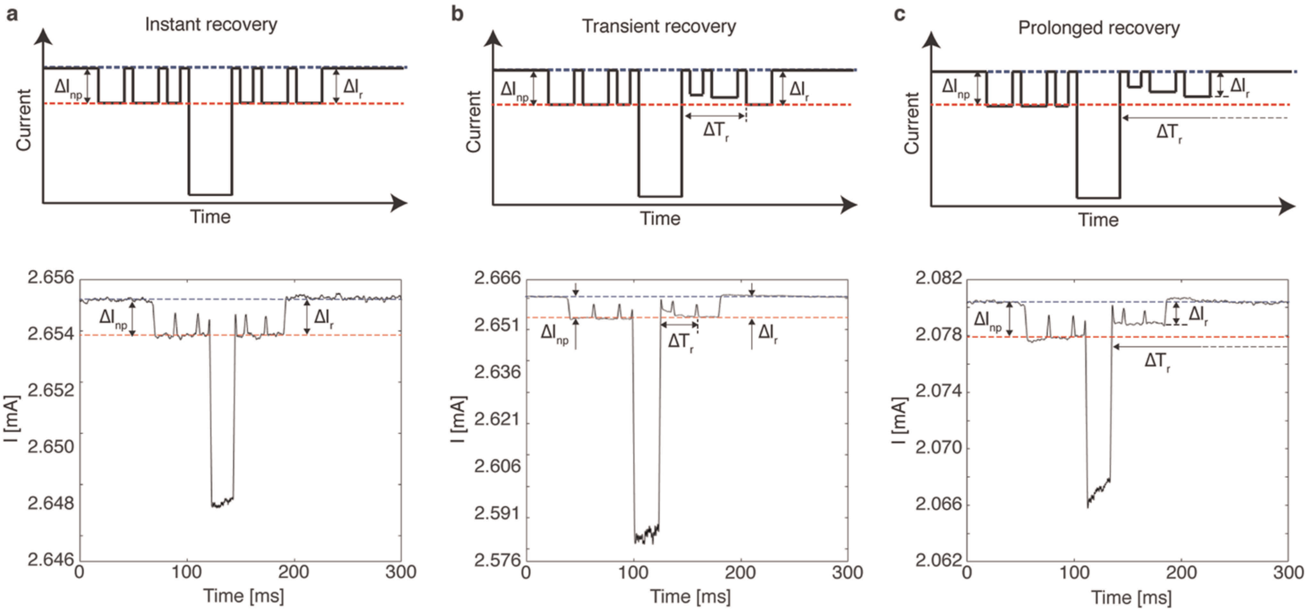

Figure S6 Schematic and representative mechano-sensing current pulses produced by an HMEC to illustrate the defined cellular recovery types after compressive deformation. (**a**) Instant recovery: The current drop (red dashed line) with respect to the baseline (blue dashed line) at the node-pore before and after the contraction channel are defined as ΔI_{np} and ΔI_r, respectively. We define "instant recovery" when a cell recovers to its original size and shape immediately after exiting the contraction channel and $\Delta I_r = \Delta I_{np}$. In this case, $\Delta T_r \sim 0$. (**b**) Transient recovery corresponds to the case when the cell recovers to its original size and shape, again defined as $\Delta I_r = \Delta I_{np}$, within the span of the node-pore sequence immediately following the contraction channel. Here, $\Delta T_r \leq 40$ ms. (**c**) Prolonged recovery corresponds to the case when the cell does not recover to its original size and shape. In this specific case, $\Delta I_r \neq \Delta I_{np}$ over the time scale recorded by mechano-NPS ($\Delta T_r > 40$ ms). All schematic drawings (**a**–**c**, top) show the idealized mechano-NPS current pulse. The representative current pulses (**a**–**c**, bottom) show that the current at the "node" does not reach to the baseline current and has a more peak-like shape. This is due to the fast flow rate of the cells and the short length of the "node" segment.

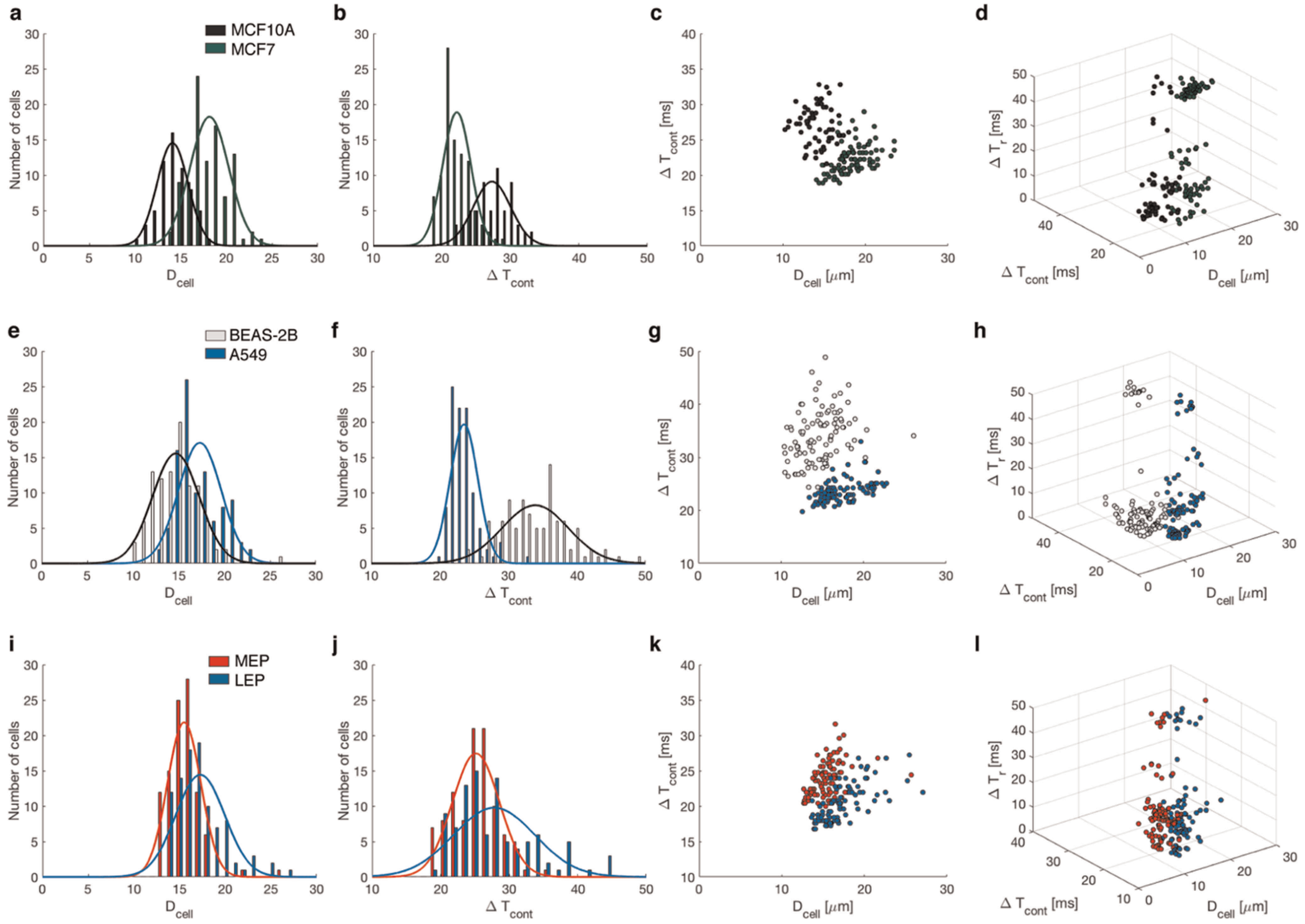

Figure S7 Biophysical parameters of breast and lung epithelial cell lines and human mammary epithelial cells as measured by mechano-NPS. (**a**) the free-cell diameter (D_{cell}) and (**b**) the transit time (ΔT_{cont}) for breast epithelial cells ($n = 99$ for both MCF-10A and MCF-7). The overlap area of the two ΔT_{cont} distributions is 28% of the total area. (**c**) ΔT_{cont} of MCF-10A and MCF-7 cells with respect to cell size. (**d**) 3D plot of the measured biophysical parameters of MCF-10A and MCF-7 cells. (**e** and **f**) Histogram of (**e**) the free-cell diameter (D_{cell}) and (**f**), ΔT_{cont} for lung epithelial cells ($n = 100$ for both BEAS-2B and A549). The overlap area of the two ΔT_{cont} distribution is 12% of the total area. (**g**) ΔT_{cont} of BEAS-2B and A549 cells with respect to cell size. (**h**) 3D plot of the measured biophysical parameters of BEAS-2B and A549 cells. (**i** and **j**) Histogram of (**i**), the free-cell diameter (D_{cell}) and (**j**), ΔT_{cont} for sorted sub-lineages of human mammary epithelial cells ($n = 99$ for both myoepithelial (MEP) cells and luminal epithelial (LEP) cells). The overlap area of the two ΔT_{cont} distributions is 68% of the total area. (**k**) ΔT_{cont} of MEP and LEP cells with respect to the cell size. (**l**) 3D plot of the measured biophysical parameters of MEP and LEP cells.

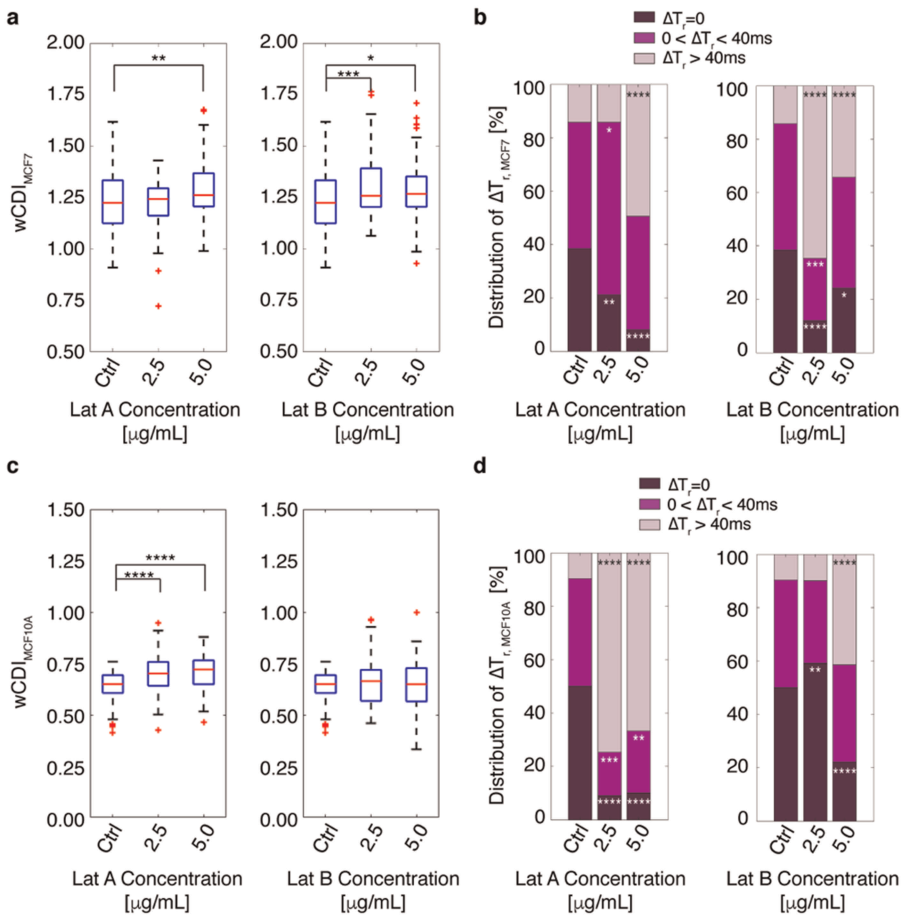

Figure S8 Mechanical phenotypes of untreated, LatA-, and LatB-treated cells. (**a**) wCDI of MCF-7 cells treated with different concentrations of LatA and LatB ($n = 100$ for all cases). Within each box, the central red line is the median, the red cross is an outlier, and the edges of the box correspond to 25% and 75% of the population. Statistical differences were determined by a paired *t*-test. (**b**) The distribution of recovery time of LatA- and LatB- treated MCF-7 cells. Statistical differences are determined by a chi-square test. (**c**) wCDI of MCF-10A cells treated with different concentrations of LatA and LatB ($n = 100$ for all cases). Within each box, the central red line is the median, the red cross is an outlier, and the edges of the box correspond to 25% and 75% of the population. Statistical differences were determined by a paired *t*-test. (**d**) The distribution of recovery time of LatA- and LatB- treated MCF-10A cells. Statistical differences are determined by a chi-square test. For all graphs, *, **, ***, and **** indicate $P \leq 0.05$, $P \leq 0.01$, $P \leq 0.001$, and $P \leq 0.0001$, respectively.

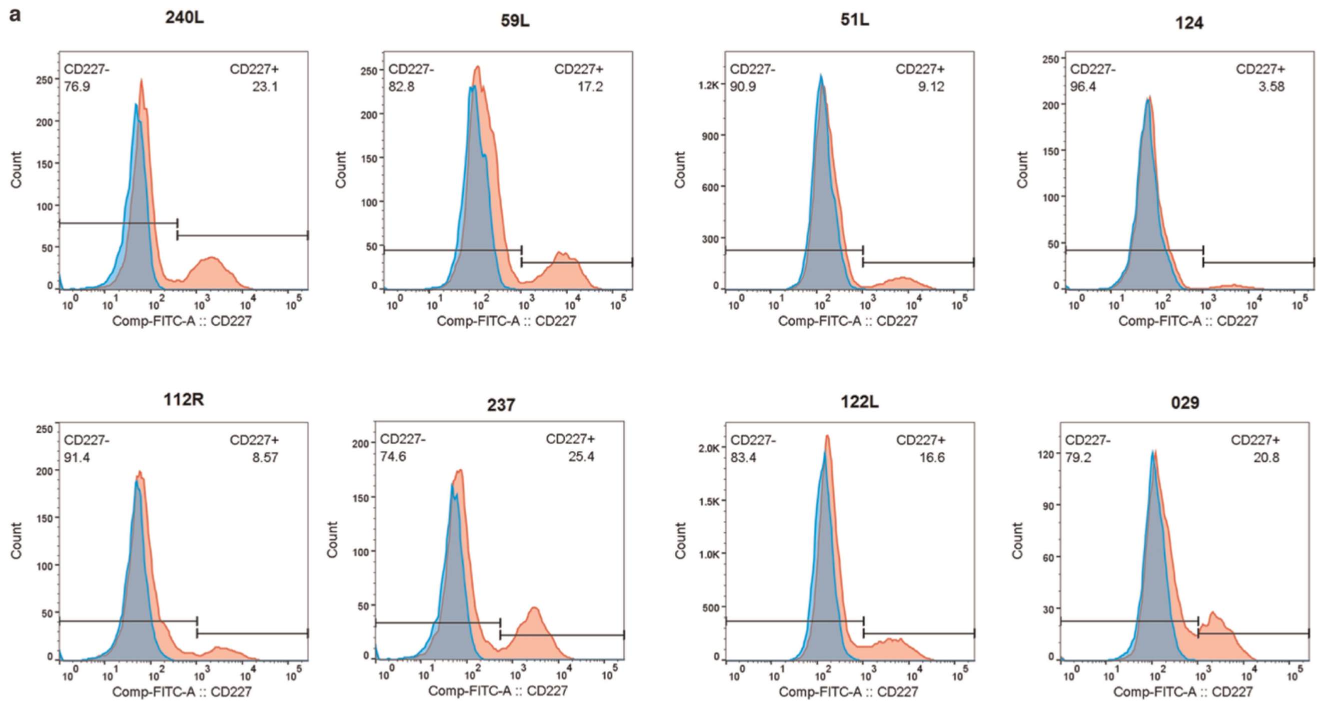

Figure S9 Fluorescence-activated cell sorting (FACS) analysis of HMECs. Representative histograms of MEP and LEP cells based on CD227 expression in different primary HMEC-strain populations after the fourth passage. Blue- and red-colored histograms correspond to the unstained negative control and CD227-stained cells, respectively (FITC, mouse anti-human CD227, Clone HMPV, BD Biosciences 559774). The component ratios of MEP and LEP cells, as determined by FACS, match exceptionally well with those obtained from the wCDI distributions, as confirmed by a chi-square test with a *P*-value = 0.05 (Supplementary Table S3).

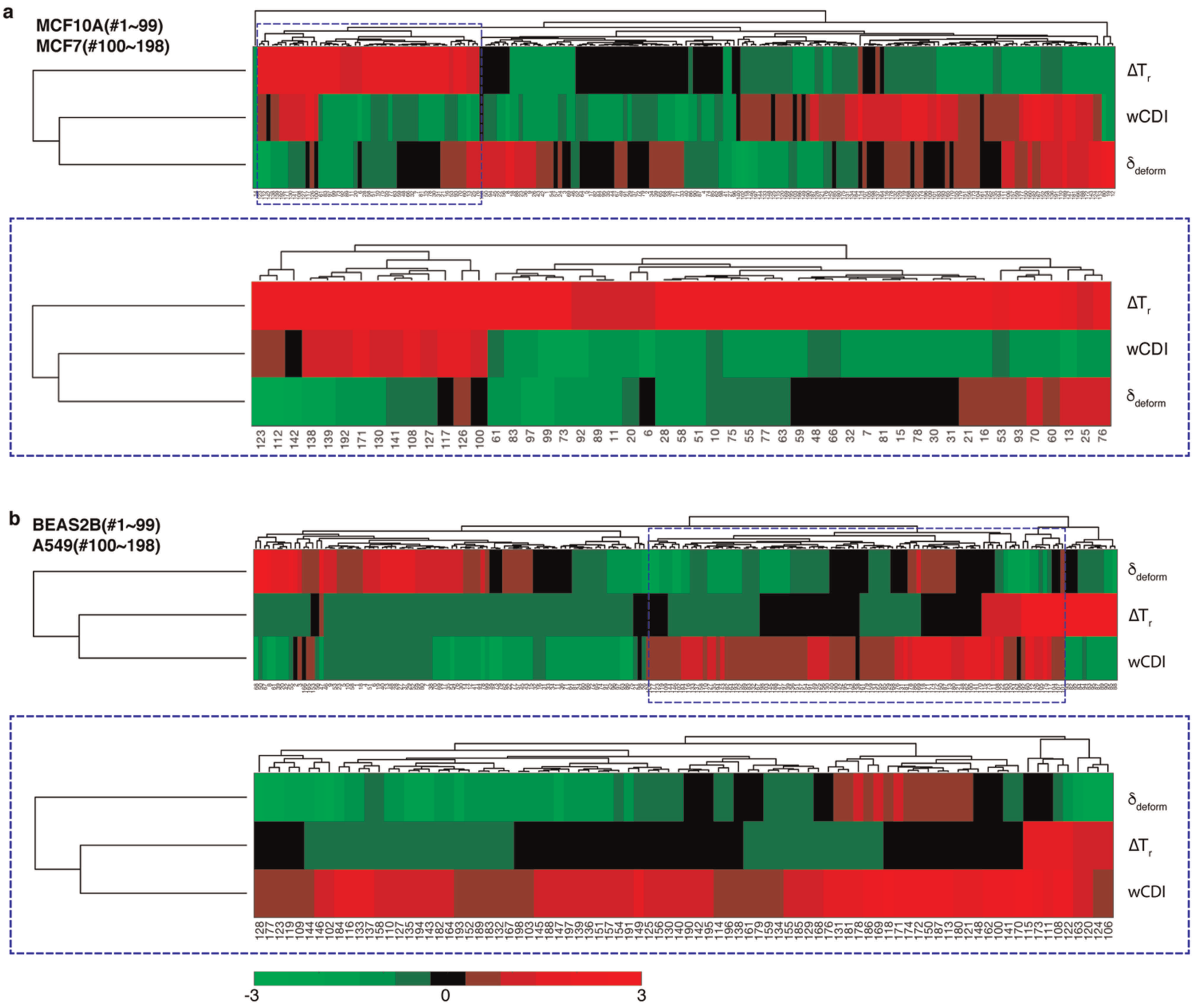

Figure S10 Hierarchical relationship among the mechanical phenotypes of breast and lung epithelial cells. (**a**) Mechanical profiling of non-malignant and malignant breast epithelial cells (MCF-10A and MCF-7, respectively). An individual column represents relative intensity of mechanical phenotypes of each single cell. Blue dashed box (bottom) shows an enlarged heat map of a sub-group of the entire population. Among the mechanical phenotypes, the *wCDI* of breast epithelial cells is more related with δ_{deform} rather than ΔT_r. (**b**) Mechanical profiling of non-malignant and malignant lung epithelial cells (BEAS-2B and A549, respectively). An individual column represents relative intensity of mechanical phenotypes of each single cell. Blue dashed box (bottom) shows an enlarged heat map of a sub-group of the entire population. Among the mechanical phenotypes, wCDI of lung epithelial cells is more related with ΔT_r rather than δ_{deform}.

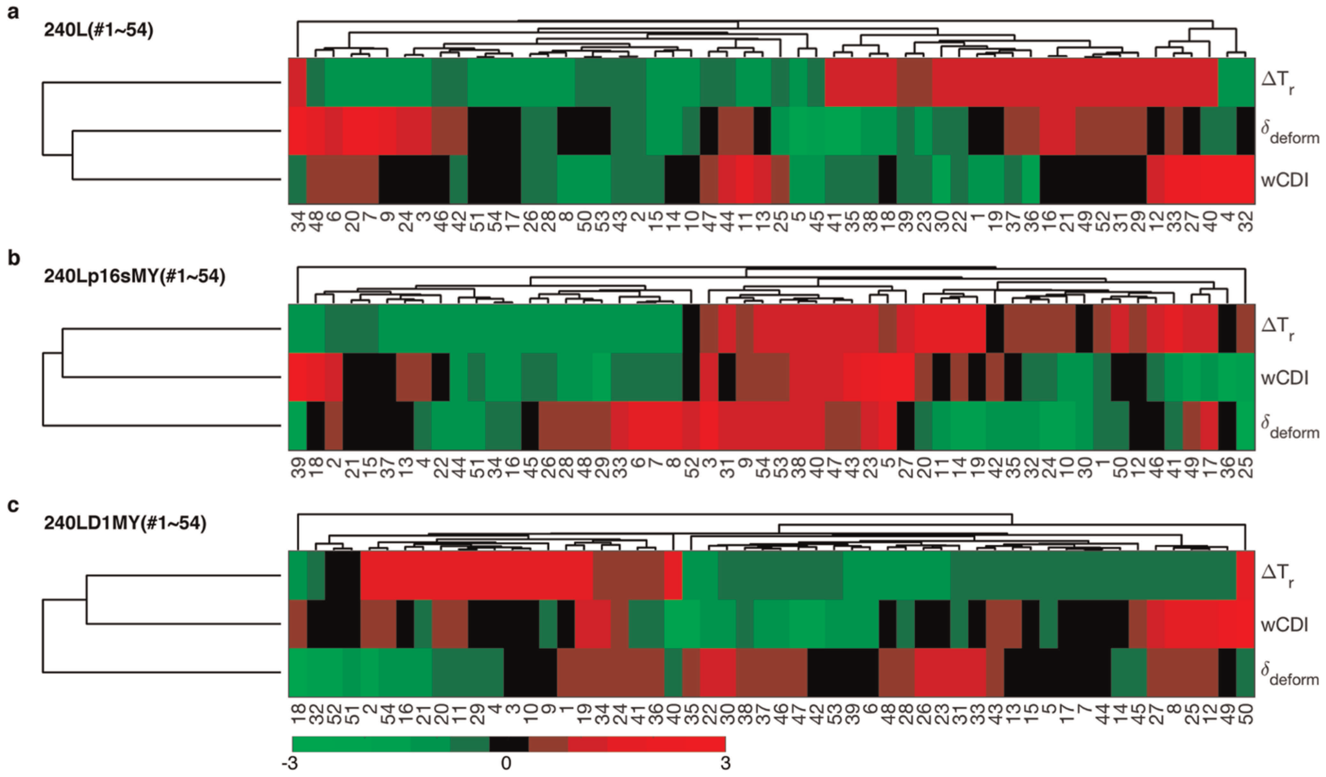

Figure S11 Hierarchical relationship among the mechanical phenotypes of pre-stasis and post-stasis human mammary epithelial cells (HMEC). (**a**) Mechanical profiling of primary HMECs (240L). An individual column represents relative intensity of mechanical phenotypes of each single cell. Among the mechanical phenotypes, the wCDI of 240L cells is more related with δ_{deform} rather than ΔT_r. (**b** and **c**) Mechanical profiling of immortalized HMECs (240Lp16sMY and 240LD1MY). An individual column represents relative intensity of mechanical phenotypes of each single cell. For the both of post stasis HMECs, the wCDI is more related with ΔT_r rather than δ_{deform}.

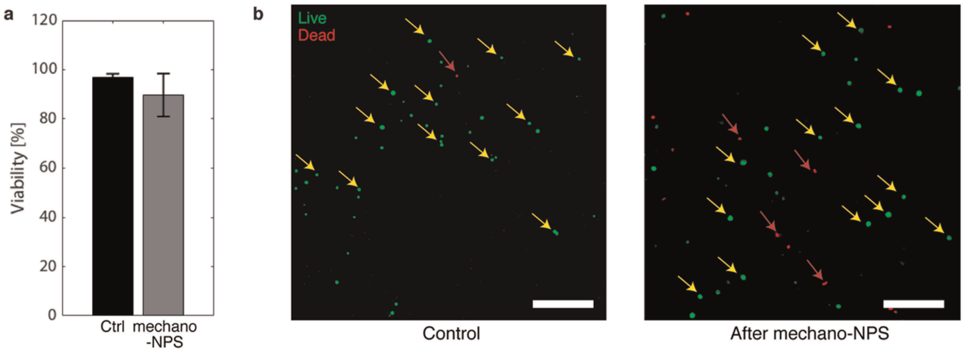

Figure S12 Cell viability after mechano-NPS screening. (**a**) Viability of A549 cells after being screened with a 0.45-magnitude strain magnitude. The control corresponds to unscreened A549 cells kept at 4 °C throughout the time the other cells were screened. All error bars are expressed as standard deviation. (**b**) Fluorescence images of control and screened cells after Live/Dead assay (LIVE/DEAD® Viability/Cytotoxicity Kit, ThermoFisher, L-3224). Green corresponds to live cells (yellow arrows) and red to dead cells (red arrows). Scale bar corresponds to 200 µm.

Table S1 Power analysis of experimental groups based on sample size

Cell type	MCF10A	MCF7	A549	BEAS-2B	MCF7_PDL	MCF7_BSA
N_a	99	99	100	100	99	99
power	1	1	1	1	0.96	0.83
Cell type	**MCF7_LatA**	**MCF7_LatB**	**MCF10A_LatA**	**MCF10A_LatB**	**MEP**	**LEP**
N_a	99	99	99	99	99	104
power	0.86	0.81	0.99	N.A.	1	1
Cell type	**240L**	**240L-p16sh**	**240Lp16sMY**	**240L-D1**	**240LD1MY**	
N_a	54	54	54	54	54	
power	0.87	N.A.	0.99	0.98	0.87	
Cell type	**122L**	**122L-p16sh**	**122Lp16sMY**	**122L-D1**	**122LD1MY**	
N_a	54	54	54	54	54	
power	1	0.83	N.A.	0.80	0.84	

To ensure adequate power to detect differences within experimental groups, we measured the power of each group using 2-sample and 1-sided power analysis with 95% confidence interval. The analyzed sample size, N_a, provided the adequate power value (≥0.80) throughout the all experimental cases. In this table, N.A indicates power analysis is not applicable due to the high *P*-value ($P \geq 0.05$).

Table S2 Measuring the channel effective diameter using polystyrene microspheres

d_{avg} [μm]	σ_d [μm]	$\Delta I/I$	L [μm]	$D_{eff.np}$ [μm]	$D_{eff.cont}$ [μm]	σ_{eff} [μm]
14.73	1.36	3.07×10^{-4}	8230	36.2	—	0.594
6.30	0.71	2.98×10^{-4}	2055	—	22.9	0.253

Polystyrene microspheres (Polysciences, #64155) suspended in PBS were measured with our NPS platform to determine the node-pore channel's effective diameter, $D_{eff.np}$ ($n = 30$) and the effective diameter of the contraction channel $D_{eff,cont}$ ($n = 12$). The d_{avg}, σ_d, $\Delta I/I$, L, and σ_{eff} correspond to the average diameter of the microspheres, the diameter standard deviation, the ratio of the current drop to baseline current, the channel length, and the effective diameter standard deviation, respectively.

Table S3 Applied strain in the contraction channel

Cell type	D_{avg} [μm]	s	w_c [μm]	ε_{avg}
MCF-7	18.06	2.179	12	0.335
MCF-10A	15.18	1.014	10	0.341
A549	17.64	2.215	12	0.320
BEAS-2B	15.11	2.542	10	0.338

D_{avg}, σ, w_c, ε_{avg} correspond to the average free cell diameter, cell diameter standard deviation, width of the contraction channel, and the average strain value, respectively. Strain is defined as the ratio of deformation to the cellular diameter, $\varepsilon = (D_{cell}\text{-}w_c)/D_{cell}$.

STATISTICAL ANALYSIS TO COMPARE *wCDI* WITH FACS ANALYSIS FOR PRIMARY HMEC STRAINS

We employed a χ^2 test to determine whether there were any statistically significant differences between the obtained wCDI and FACS results:

$$\chi^2 = \sum_{i=1}^{n} \frac{(O_i \quad E_i)^2}{E_i} \qquad \text{(S1)}$$

The observed values, O_i, and expected values E_i, were the number of MEP and LEP cells as measured by mechano-NPS and FACS, respectively. Supplementary Table 3 shows the χ^2 values for the different HMEC strains. For a *P*-value = 0.05, $\chi^2 = 3.841$. Thus, there are no statistically significant differences between mechano-NPS and FACS.

Table S4 Chi-square (χ^2) score of HMEC strains comparing wCDI with FACS analysis

Strain	240L	59L	51L	124	112R	237	122L	29
MEP, χ^2	0.242	0.368	0.361	0.060	0.037	0.618	0.127	0.384
LEP, χ^2	0.805	1.769	3.615	1.634	0.386	1.814	0.881	1.463

REFERENCES

1 Saleh OA. *A novel resistive pulse sensor for biological measurements*. Princeton: Princeton University, PhD Thesis 2003.

2 Carbonaro A, Sohn L. A resistive-pulse sensor chip for multianalyte immunoassays. *Lab on a Chip* 2005; **5**: 1155–1160.

3 Dokukin ME, Guz NV, Sokolov I. Quantitative study of the elastic modulus of loosely attached cells in AFM indentation experiments. *Biophysical journal* 2013; **104**: 2123–2131.

4 Li Q, Lee G, Ong C *et al*. *Probing the elasticity of breast cancer cells using AFM. 13th International Conference on Biomedical Engineering; 2009*. Springer. 2009 pp 2122–2125.

5 Rother J, Nöding H, Mey I *et al.* Atomic force microscopy-based microrheology reveals significant differences in the viscoelastic response between malign and benign cell lines. *Open biology* 2014; **4**: 140046.
6 Li Q, Lee G, Ong C *et al.* AFM indentation study of breast cancer cells. *Biochemical and biophysical research communications* 2008; **374**: 609–613.
7 Xu C, Wang Y, Jiang N *et al.* Elasticity measurement of breast cancer cells by atomic force microscopy*Twelfth International Conference on Photonics and Imaging in Biology and Medicine (PIBM 2014); 2014*. International Society for Optics and Photonics. 2014 pp. 92300Y-92300Y-92306.
8 Alcaraz J, Buscemi L, Grabulosa M *et al.* Microrheology of human lung epithelial cells measured by atomic force microscopy. *Biophysical journal* 2003; **84**: 2071–2079.
9 Acerbi I, Luque T, Giménez A *et al.* Integrin-specific mechanoresponses to compression and extension probed by cylindrical flat-ended AFM tips in lung cells. *PLoS One* 2012; **7**: e32261.
10 Demichelis A, Divieto C, Mortati L *et al.* *Preliminary measurements of elasticity properties of lung tumor living cells for cancer detection. Medical Measurements and Applications (MeMeA), 2015 IEEE International Symposium on; 2015*. IEEE. 2015 pp 240–243.

This work is licensed under a Creative Commons Attribution 4.0 International License. The images or other third party material in this article are included in the article's Creative Commons license, unless indicated otherwise in the credit line; if the material is not included under the Creative Commons license, users will need to obtain permission from the license holder to reproduce the material. To view a copy of this license, visit http://creativecommons.org/licenses/by/4.0/

© The Author(s) 2018
